# *Helicobacter pylori* antibiotic resistance genes and virulence based on fecal samples in Qinghai, China: A cross-sectional study

**DOI:** 10.1097/MD.0000000000049935

**Published:** 2026-07-24

**Authors:** Shenghui Wei, Xuehong Wang, Ling Xu

**Affiliations:** a School of Clinical Medicine, Qinghai University, Xining, Qinghai, China; b Department of Gastroenterology, Affiliated Hospital of Qinghai University, Xining, Qinghai, China.

**Keywords:** antibiotic resistance genes, *Helicobacter pylori*, virulence

## Abstract

Antibiotic resistance in *Helicobacter pylori (H pylori*) is influenced by multiple factors and shows significant geographical variation. Qinghai has a high incidence of gastric cancer and a high prevalence of *H pylori* infection, which may be exacerbated by factors such as limited healthcare resources, an underdeveloped surveillance system, a challenging geographical environment and climate, and distinct local lifestyle habits. This study aimed to investigate the distribution of resistance genes and virulence factors of *H pylori* strains isolated from patients in the Qinghai region to levofloxacin, clarithromycin, amoxicillin, tetracycline, and furazolidone, in order to provide a scientific basis for the precise treatment of *H pylori* in Qinghai. Between October 2024 and August 2025, 151 patients in Qinghai province who underwent ^13^C-urea breath testing for *H pylori* infection were enrolled. The overall antibiotic resistance rate of *H pylori* in Qinghai province was 80.60%, with the highest rate observed for clarithromycin (58.21%) and the lowest for tetracycline (14.18%). Resistance rates for levofloxacin, amoxicillin, and furazolidone were 41.04%, 17.91%, and 17.91%, respectively. The rates of dual and multidrug resistance were 31.34% and 17.91%, respectively. Antibiotic resistance in *H pylori* from Qinghai poses a significant challenge, with a concomitantly high detection rate of virulence factors. Therefore, it is imperative to proactively implement antibiotic susceptibility testing and assess relevant influence factors for *H pylori*-infected patients in Qinghai prior to the 1st eradication therapy to reduce antibiotic resistance.

## 1. Introduction

*Helicobacter pylori (H pylori*) is a microaerophilic, gram-negative bacterium that colonizes the human gastric mucosa,^[1]^ and it is highly contagious. Epidemiological studies indicate that *H pylori* is one of the most prevalent bacterial infections globally, particularly in developing countries,^[2]^ and is one of the primary etiological agents of gastrointestinal diseases such as chronic gastritis, peptic ulcers, intestinal metaplasia, and gastric cancer.^[3]^ Therefore, as early as 1994, *H pylori* was classified as a group I biological carcinogen by the WHO.^[4]^ Although the global *H pylori* infection rate has been declining annually in recent years, it remains as high as 50%. The infection rate is 44.2% in inland China and 52.8% in Qinghai province, which is substantially higher than the national and global average.^[5]^ Current guidelines indicate that all *H pylori*-positive patients should receive eradication therapy in the absence of contraindications.^[6]^ Although there are various regimens for *H pylori* eradication, the widespread use of antibiotics has led to progressively increasing resistance, which has emerged as a primary cause of empirical eradication failure.^[7]^ Studies have shown that an initial eradication failure or a history of multiple eradication attempts using the same drug makes individuals more susceptible to developing resistant strains.^[8]^ Domestic and international guidelines concur that prior knowledge of antibiotic susceptibility is crucial for successful eradication.^[9]^

Antibiotic resistance in *H pylori* is influenced by multiple factors and shows significant geographical variation.^[10,11]^ The Qinghai–Tibet Plateau primarily comprises the Tibet Autonomous Region and Qinghai province. Qinghai has a high incidence of gastric cancer and a high prevalence of *H pylori* infection, which may be exacerbated by factors such as limited healthcare resources, an underdeveloped surveillance system, a challenging geographical environment and climate, and distinct local lifestyle habits.^[5]^ Quantitative polymerase chain reaction (qPCR) is an emerging technique for antibiotic resistance testing,^[6]^ while fecal qPCR, with its advantages of being noninvasive, convenient, well-accepted, and highly specific, has been implemented in some regions. Currently, research on *H pylori* resistance genes in fecal samples from the Qinghai region remains limited. Undertaking fecal-based qPCR testing for antibiotic resistance genes in this area is of significant scientific and practical importance as it can provide crucial data support for clinical treatment.

## 2. Methods

### 2.1. Study population and location

A total of 151 patients with positive *H pylori* infection detected by the ^13^C-urea breath test in Qinghai province between October 2024 and August 2025 were enrolled from the Department of Gastroenterology Outpatient Clinic, Affiliated Hospital of Qinghai University. A consecutive enrollment method was adopted, and the sample size was determined by the actual number of patients presenting during the study period. Among them, 100 patients had never received eradication therapy for *H pylori*, and 51 patients had experienced initial eradication failure. Basic patient information was obtained from the electronic medical record system, including age, sex, residential area, smoking status, alcohol and tea consumption, frequency of dining out, and clinical symptoms.

### 2.2. Inclusion criteria

Patients with positive *H pylori* results on the ^13^C-urea breath test and no relevant contraindications (e.g., antibiotic allergy, severe hepatic, or renal insufficiency), who provided informed consent for resistance gene testing, were capable of providing fecal samples meeting the trial’s required sample volume, and patients aged 18 to 75 years, regardless of sex.

### 2.3. Exclusion criteria

The use of *H pylori*-sensitive medications within the past month, including antibiotics, bismuth agents, proton pump inhibitors, potassium-competitive acid blockers, or anti-*H pylori* Chinese herbal medicine; presence of recent infection, diarrhea, or severe constipation; and inability to obtain a sufficient and qualified fecal sample.

### 2.4. *H pylori* testing

*H pylori* infection was confirmed by the ^13^C-urea breath test. A delta over baseline value of 4.0 was set as the cutoff point; a value ≥4.0 was defined as *H pylori*-positive, confirming infection, while a value <4.0 was defined as *H pylori*-negative, indicating no infection.

### 2.5. Sample collection and processing

Patients with positive *H pylori* infection were instructed to collect 3 to 5 g of stool using a designated collection tool after morning bowel movement. Each sample was placed in a collection tube containing a specialized preservation solution, labeled with the corresponding sample information, and stored at room temperature for subsequent submission and testing.

### 2.6. Detection of *H. pylori* and antibiotic resistance genes in fecal samples

Testing was performed by Jiangsu Cowin Biotech Co., Ltd., an independent 3rd-party testing company, according to the manufacturer’s instructions. Mutation detection kits targeting *H pylori* gyrA, 23S rRNA, pbp1A, 16S rRNA, and porD were used in this study. PCR melting-curve analysis identified mutation genotypes in these loci to determine the resistance and mutation sites of levofloxacin (LVFX), clarithromycin (CLA), amoxicillin (AMX), tetracycline (TC), and furazolidone (FZD). The PCR system included 29 μL of *H pylori*-PCRmaster mix, 1 μL of DNA polymerase, and 20 μL of the extracted DNA sample. The PCR master mix contained primers and probes for gyrA, 23S rRNA, pbp1A, 16S rRNA, porD, ACTB genes, dNTPs, ddH_2_O, and a Tris-HCl buffer system.

Melting-curve analysis was conducted to discriminate between wild-type and mutant strains based on the melting temperature (Tm) shifts. Specimens displaying Tm values consistent with the wild-type control were designated as susceptible, whereas those showing Tm shifts were considered resistant. The specific mutation sites detected and their corresponding resistance types are detailed in the Table 1. As the assay kit employed in the present study is a commercially available product, the primer sequences, specific annealing temperature, thermal cycling conditions, and fluorescence threshold settings constitute proprietary information of the manufacturer and are not publicly available; consequently, they are not elaborated upon in this manuscript.

**Table 1:** Specific mutation sites and their corresponding resistance types.

Antibiotic	Antibiotic resistance gene mutation sites
LVFX (gyrA)	N87I, N87K, D91N, D91Y, D91G
CLA (23S rRNA)	A2142G, A2142C, A2143G
AMX (pbp1A)	T593A, S414R, T556S
TC (16S rRNA)	A926G, A928C, A926T
FZD (porD)	G343A, G353A, A346G, C347T, A357T

AMX = amoxicillin, CLA = clarithromycin, FZD = furazolidone, LVFX = levofloxacin, TC = tetracycline.

### 2.7. Statistical methods

Statistical analyses were performed using SPSS version 26.0 (IBM Corp., Armonk). All figures were generated using the GraphPad Prism version 9.4.0 software (GraphPad Software, San Diego). Categorical data were presented as numbers and percentages. The effects of influence factors on antibiotic resistance rates were evaluated using chi-square (χ^2^) and Fisher exact tests. Differences in *H pylori* resistance genes and virulence between the 2 patient groups were further analyzed. Statistical significance was set at *P* < .05.

### 2.8. Ethics

This study was approved by the Medical Ethics Committee of Qinghai University Affiliated Hospital (P-SL-2024-251). Written informed consent was obtained from all participants after they were fully briefed about the study and their rights.

## 3. Results

### 3.1. Antibiotic resistance rates and patterns among *H pylori*-infected patients in Qinghai province

**Figure 1.**
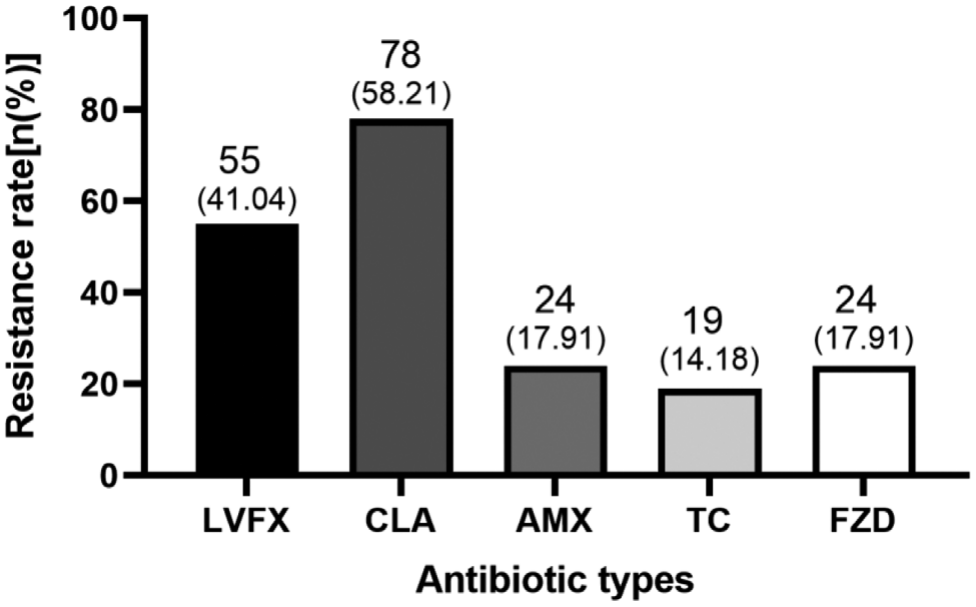
Antibiotic resistance rate. AMX = amoxicillin, CLA = clarithromycin, FZD = furazolidone, LVFX = levofloxacin, TC = tetracycline.

The fecal *H pylori* nucleic acid test showed a positivity rate of 88.74% (134/151), and the remaining 17 were negative. The overall antibiotic resistance rate of *H pylori* was 80.60% (108/134), with CLA exhibiting the highest resistance rate at 58.21% (78/134), whereas TC exhibited the lowest resistance rate at 14.18% (19/134). The overall resistance rates to LVFX, AMX, and FZD were 41.04% (55/134), 17.91% (24/134), and 17.91% (24/134), respectively (Fig. 1). The rates of dual and multidrug resistance were 31.34% (42/134) and 17.91% (24/134), respectively.

The LVFX-plus-CLA combination was the predominant dual-drug resistance pattern in this study, accounting for 50.00% (21/42) of all cases, while the proportions of other patterns were relatively low (Table 2). The predominant multidrug resistance patterns were LVFX, CLA, and AMX, accounting for 45.83% (11/24) of all resistance patterns, while the proportions of the remaining patterns were relatively low (Table 3).

**Table 2:** Proportion of dual-drug resistance patterns (n [%]).

Antibiotics	n	Antibiotics	n
LVFX + CLA	21 (50.00)	TC + FZD	2 (4.76)
LVFX + FZD	6 (14.29)	CLA + FZD	1 (2.38)
CLA + TC	5 (11.91)	LVFX + TC	1 (2.38)
CLA + AMX	3 (7.14)	LVFX + AMX	1 (2.38)
AMX + FZD	2 (4.76)		

AMX = amoxicillin, CLA = clarithromycin, FZD = furazolidone, LVFX = levofloxacin, TC = tetracycline.

**Table 3:** Proportion of multidrug resistance patterns (n [%]).

Antibiotics	n	Antibiotics	n
LVFX + CLA + AMX	11 (45.83)	CLA + TC + FZD	1 (4.17)
LVFX + CLA + TC	3 (12.50)	CLA + AMX + TC	1 (4.17)
LVFX + CLA + FZD	2 (8.33)	LVFX + AMX + TC	1 (4.17)
CLA + AMX + FZD	2 (8.33)	LVFX + CLA + TC + FZD	1 (4.17)
LVFX + TC + FZD	1 (4.17)	LVFX + CLA + AMX + TC	1 (4.17)

AMX = amoxicillin, CLA = clarithromycin, FZD = furazolidone, LVFX = levofloxacin, TC = tetracycline.

### 3.2. Antibiotic resistance gene profile in *H pylori*-infected patients in Qinghai province

**Figure 2.**
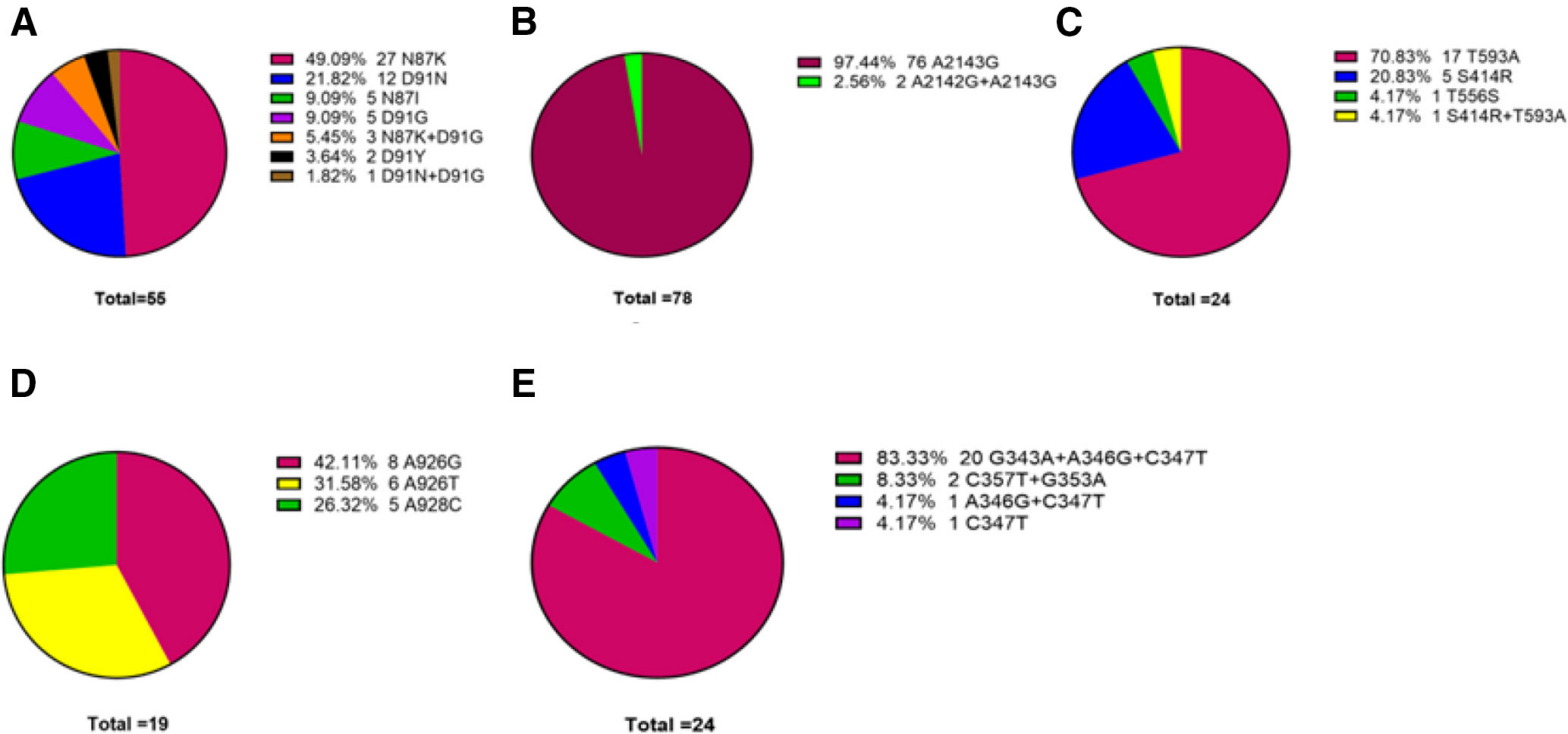
Antibiotic resistance gene profile in *Helicobacter pylori*-infected patients in Qinghai province. (A) Genomic profile of LVFX resistance. (B) Genomic profile of CLA resistance. (C) Genomic profile of AMX resistance. (D) Genomic profile of TC resistance. (E) Genomic profile of FZD resistance. AMX = amoxicillin, CLA = clarithromycin, FZD = furazolidone, LVFX = levofloxacin, TC = tetracycline.

The predominant mutation site for LVFX resistance was N87K, accounting for 49.09% (27/55) of the mutations, while the remaining mutations accounted for a relatively small proportion (Fig. 2A). The predominant mutation site for CLA resistance was A2143G, accounting for 97.44% (76/78) (Fig. 2B). The predominant mutation site for AMX resistance was T593A, accounting for 70.83% (17/24) of the mutations, while the prevalence of other mutations was relatively low (Fig. 2C). The predominant mutation site for TC resistance was A926G, accounting for 42.11% (8/19), whereas A926T and A928C accounted for 31.58% and 26.32%, respectively (Fig. 2D). The predominant mutation pattern for FZD resistance was G343A + A346G + C347T, accounting for 83.33% (20/24) (Fig. 2E).

### 3.3. Detection of virulence factors in fecal *H pylori* nucleic acid testing

**Figure 3.**
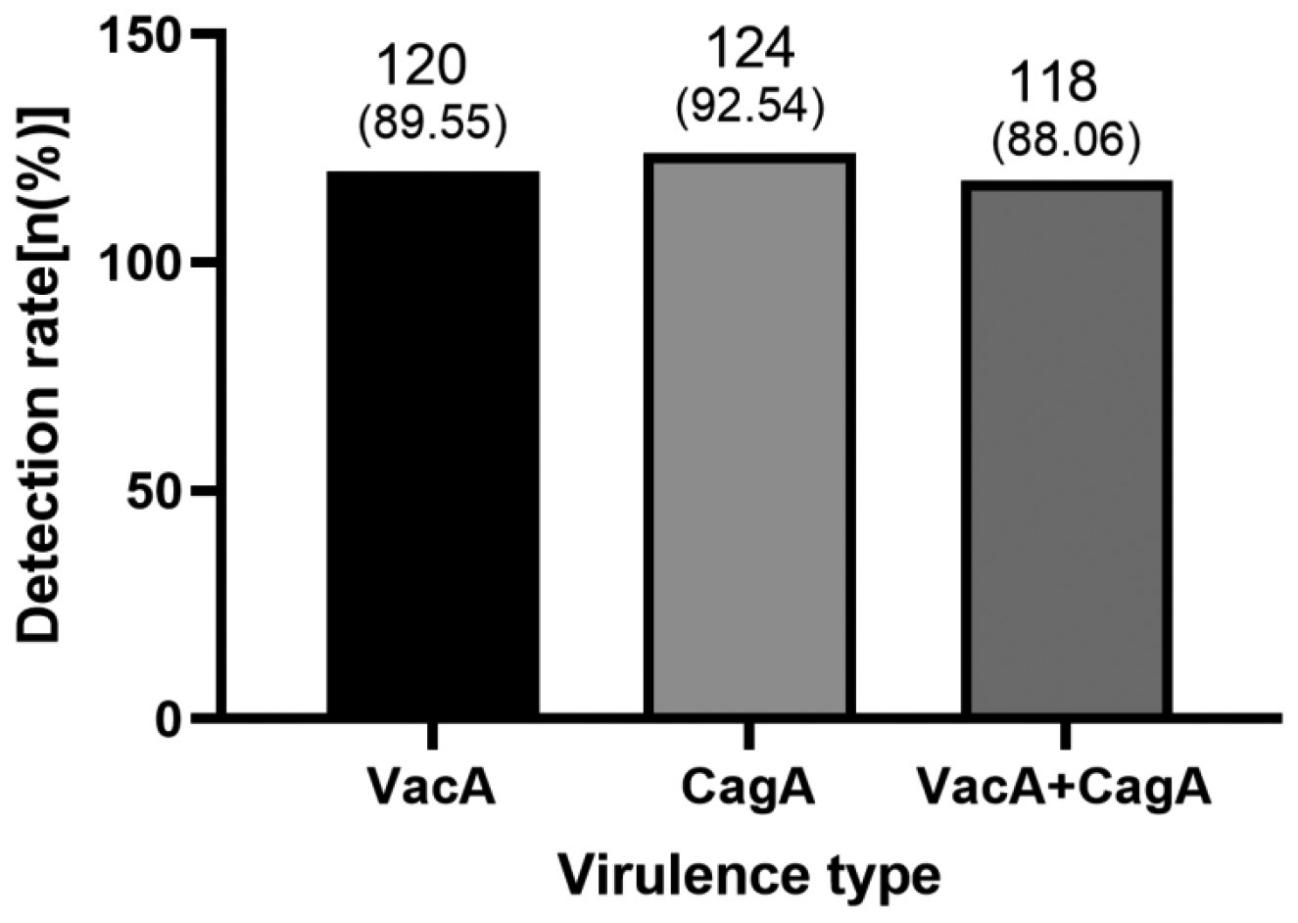
Detection rate of virulence factors. CagA = cytotoxin-associated gene A, VacA = vacuolating toxin gene A.

The overall detection rates for the virulence factors vacuolating toxin gene A (VacA) and cytotoxin-associated gene A (CagA) were 89.55% (120/134) and 92.54% (124/134), respectively. The detection rate of dual virulence factors (VacA + CagA) was 88.06% (118/134) (Fig. 3).

### 3.4. Impact of different influencing factors on *H pylori* resistance rates

This study included 76 males and 75 females, with an age range of 18 to 75 years (mean ± standard deviation: 46.09 ± 13.275). Among them, 134 patients (66 males and 68 females) tested positive for fecal *H pylori* nucleic acid and were stratified by age as follows: 117 patients aged 18 to 59 years and 17 patients aged 60 to 75 years.

The resistance rate to LVFX was relatively higher among patients aged 60 years and above; female patients exhibited a higher resistance rate to CLA, while smokers showed a higher resistance rate to AMX, and patients who frequently dined out demonstrated a higher resistance rate to TC. In contrast, none of the influencing factors examined exerted a significant effect on resistance to FZD (Table 4).

**Table 4:** The influence factors on antibiotic resistance (n [%]).

Antibiotics	Influence factors	Groups	Drug resistant (n)	Not resistant (n)	χ^2^	*P*
LVFX	Age	<60	42 (31.34)	75 (55.97)	10.10	<.01
		≥60	13 (9.70)	4 (2.99)		
CLA	Gender	Male	29 (21.64)	37 (27.61)	10.89	<.01
		Female	49 (36.57)	19 (14.18)		
AMX	Smoking status	No	11 (8.21)	77 (57.46)	5.10	.02
		Yes	13 (9.70)	33 (24.63)		
TC	Dining out	Occasionally	4 (2.99)	53 (39.55)	4.18	.04
		Frequently	15 (11.19)	62 (46.27)		

Regarding eating out, eating <2 times per week was considered occasional, while eating ≥2 times per week was considered frequent.

AMX = amoxicillin, CLA = clarithromycin, LVFX = levofloxacin, TC = tetracycline.

### 3.5. Comparison of antibiotic resistance rates and virulence factors between the 2 groups

To further analyze the status of antibiotic resistance rates and virulence factors between the 2 groups, 134 patients were stratified into a never-treated group A (group A, n = 96) and an initial eradication failure group (group B, n = 38). In group B, the resistance rates to CLA and AMX were significantly higher than those in group A (*P *< .05). However, the resistance rates to LVFX, TC, and FZD were not significantly different between the 2 groups (Table 5).

**Table 5:** Comparison of antibiotic resistance rates and virulence factors between the 2 groups (n [%]).

Antibiotics	Groups	Drug resistance	χ^2^	*P*
LVFX	Group A	37 (38.54)	0.87	.35
	Group B	18 (47.37)		
CLA	Group A	50 (52.08)	5.22	.02
	Group B	28 (73.68)		
AMX	Group A	12 (12.50)	5.49	.02
	Group B	12 (31.58)		
TC	Group A	12 (12.50)	0.62	.39
	Group B	7 (18.42)		
FZD	Group A	20 (20.83)	1.33	.15
	Group B	4 (10.53)		

AMX = amoxicillin, CLA = clarithromycin, FZD = furazolidone, LVFX = levofloxacin, TC = tetracycline.

For both dual and multidrug resistance, the resistance rates in group B were significantly higher than those in group A. The difference in the multidrug resistance rate was statistically significant (*P* < .05) (Table 6).

**Table 6:** Detection of dual and multidrug resistance (n [%]).

Resistance patterns	Groups	n	χ^2^	*P*
Dual-drug resistance	Group A	28 (29.17)	0.43	.51
	Group B	14 (36.84)		
Multidrug resistance	Group A	13 (13.54)	3.40	.04
	Group B	11 (28.95)		

In group B, the detection rates of the virulence factors VacA, CagA, and the dual factor (VacA + CagA) were higher than those in group A, although the differences were not statistically significant (*P* > .05) (Table 7).

**Table 7:** Detection of virulence factors (n [%]).

Virulence factor		Group A (n = 96)	Group B (n = 38)	χ^2^	*P*
VacA	+	85 (88.54)	35 (92.11)	0.37	.48
	−	11 (11.46)	3 (7.89)		
CagA	+	88 (91.67)	36 (94.74)	0.06	.68
	−	8 (8.33)	2 (5.26)		
VacA + CagA	+	84 (87.50)	34 (89.47)	<0.01	1.00
	−	12 (12.50)	4 (10.53)		

CagA = Cytotoxin-associated gene A, VacA = Vacuolating toxin gene A.

### 3.6. Comparison of antibiotic resistance gene profiles between the 2 groups

The differences between the various resistance gene mutation sites were analyzed in detail. For the CLA resistance gene, the A2143G mutation was the predominant site in both groups, accounting for 96.00% in group A and 100% in group B. For the AMX resistance gene, the T593A mutation was the predominant site in both groups, accounting for 75.00% in group A and 66.67% in group B; the remaining mutation sites accounted for a relatively small proportion. None of the differences in resistance gene mutation sites between the groups were statistically significant (*P* > .05) (Table 8).

**Table 8:** Differences in the antibiotics resistance gene spectrum (n [%]).

Drug resistance gene	Groups	n	χ^2^	*P*
A2143G	Group A	48 (96.00)	0.11	.53
	Group B	28 (100)		
A2142G + A2143G	Group A	2 (4.00)	0.11	.53
	Group B	0 (0)		
T593A	Group A	9 (75.00)	0.00	1.00
	Group B	8 (66.67)		
S414R	Group A	1 (8.33)	1.01	.32
	Group B	4 (33.33)		
T556S	Group A	1 (8.33)	0.00	1.00
	Group B	0 (0)		
S414R + T593A	Group A	1 (8.33)	0.00	1.00
	Group B	0 (0)		

### 3.7. Comparison of resistance patterns between the 2 groups

In both groups, the LVFX-plus-CLA combination showed the predominant dual-drug resistance pattern. This pattern accounted for 46.43% (13/28) in group A and 57.14% (8/14) in group B, whereas the remaining patterns accounted for relatively low proportions in both groups (Table 9).

**Table 9:** Proportion of dual-drug resistance patterns (n [%]).

Resistance patterns	Group A (n = 28)	Resistance patterns	Group B (n = 14)
LVFX + CLA	13 (46.43)	LVFX + CLA	8 (57.14)
LVFX + FZD	5 (17.86)	CLA + TC	2 (14.29)
CLA + TC	3 (10.71)	TC + FZD	1 (7.14)
CLA + AMX	2 (7.14)	LVFX + FZD	1 (7.14)
AMX + FZD	2 (7.14)	CLA + AMX	1 (7.14)
TC + FZD	1 (3.57)	CLA + FZD	1 (7.14)
LVFX + TC	1 (3.57)		
LVFX + AMX	1 (3.57)		

AMX = amoxicillin, CLA = clarithromycin, FZD = furazolidone, LVFX = levofloxacin, TC = tetracycline.

In both groups, the LVFX-plus-CLA-plus-AMX combination showed the predominant multidrug resistance pattern. It accounted for 38.46% (5/13) of the patients in group A and 54.55% (6/11) in group B, while the remaining patterns accounted for relatively low proportions (Table 10).

**Table 10:** Proportion of multidrug resistance patterns (n [%]).

Resistance patterns	Group A (n = 13)	Resistance patterns	Group B (n = 11)
LVFX + CLA + AMX	5 (38.46)	LVFX + CLA + AMX	6 (54.55)
LVFX + CLA + FZD	2 (15.38)	LVFX + AMX + TC	1 (9.09)
LVFX + CLA + TC	2 (15.38)	LVFX + CLA + TC	1 (9.09)
LVFX + TC + FZD	1 (7.69)	CLA + AMX + TC	1 (9.09)
CLA + TC + FZD	1 (7.69)	CLA + AMX + FZD	1 (9.09)
CLA + AMX + FZD	1 (7.69)	LVFX + CLA + AMX + TC	1 (9.09)
LVFX + CLA + TC + FZD	1 (7.69)		

AMX = amoxicillin, CLA = clarithromycin, FZD = furazolidone, LVFX = levofloxacin, TC = tetracycline.

## 4. Discussion

Studies have shown that up to 90% of gastric cancer cases are attributable to *H pylori* infection. *H pylori* eradication can significantly reduce its incidence; as a controllable risk factor for gastric cancer, eradication of *H pylori* can significantly reduce its incidence.^[12]^ Therefore, the detection and eradication of *H pylori* are of paramount importance. Among the conventional noninvasive methods for *H pylori* detection, the sensitivities of serum antibody testing, ^13^C-urea breath test, and ^14^C-urea breath test were 84.00%, 94.00%, and 92.00%,^[13]^ respectively. In this study, the positive rate of fecal *H pylori* nucleic acid testing was 88.74%, indicating a high sensitivity. Among the patients, 17 had negative fecal nucleic acid test results, whereas their ^13^C-urea breath test results were all positive, suggesting that these negative results may be false negatives. This discrepancy may be related to bacterial factors (such as coccoid transformation) or pre-analytical factors (e.g., degradation of *H pylori* DNA or failure to sample DNA-enriched regions).

Studies have shown that the overall global antibiotic resistance rate of *H pylori* ranges from 15.0% to 50.0%.^[14]^ In this study, the overall resistance rate in Qinghai was 80.60%, which is significantly higher than the global average. This elevated rate may be associated with factors such as healthcare resources, surveillance systems, and geographical climate,^[15]^ warranting considerable attention. In the Qinghai region, *H pylori* was most susceptible to TC, followed by AMX and FZD. The resistance rates to these 3 antibiotics remain low, suggesting that they can still be recommended as 1st-line agents for *H pylori* eradication. In contrast, resistance rates to CLA and LVFX were 58.21% and 41.04%, respectively. Therefore, they are no longer suitable as 1st-line agents in the Qinghai region. However, they can be considered for precision treatment based on antimicrobial susceptibility testing. In the Nanjing region, the resistance rates to CLA and LVFX were 51.15% and 37.69%, respectively,^[16]^ which differed from those in Qinghai. This disparity highlights the importance of fecal *H pylori* antimicrobial susceptibility testing and precision therapy for fecal *H pylori*.

In this study, the rates of both dual and multidrug resistance were higher in group B than in group A. Multidrug resistance was associated with eradication failure. This indicates that unsuccessful eradication therapy can further increase the resistance rates. Therefore, it is imperative to improve the 1st-line eradication success rate and curb the increase in antibiotic resistance. Regarding resistance patterns, dual resistance was predominantly characterized by the LVFX-plus-CLA combination, whereas multidrug resistance primarily involved LVFX, CLA, and AMX. When *H pylori* develops multidrug resistance, drug selection is extremely difficult. This may lead to increased therapeutic challenges and a further increase in the risk of resistance. For patients with multidrug resistance, high-dose dual therapy may be an effective approach.^[17]^

Resistance to LVFX arises from its interference with the binding of antibiotics to DNA gyrase.^[14]^ In the present study, the primary resistance mutation site was identified as N87K. Other identified mutation sites included D91N, N87I, D91G, D91Y, and the combination of N87K + D91G and D91N + D91G. Resistance to CLA occurs because of the reduced affinity for 23S rRNA. In this study, the A2143G mutation was the predominant resistance site, and combined A2142G and A2143G mutations were also identified. Resistance to AMX develops when mutations impair cell wall synthesis.^[14]^ The mutations identified in this study included T593A, S414R, T556S, and a combination of S414R and T593A mutations. TC resistance occurs when an antibiotic prevents aminoacyl-tRNA from binding to the ribosome, thereby inhibiting protein synthesis. Studies have shown that resistance mutations are concentrated at A926C and A926T in the 16S rRNA gene.^[18]^ However, in the Qinghai region, the predominant mutations are A926G, A928C, and A926T, reflecting the complex diversity of genetic mutations. For porD mutations associated with FZD resistance in Qinghai, the predominant pattern was the combination of G343A, A346G, and C347T. A C347T mutation has also been identified. The primary LVFX resistance mutation sites in the Lianyungang area of Jiangsu province were N87K, D91Y, and D91G, accounting for 45.0%, 30.0%, and 25.0%, respectively. The main CLA resistance site was A2143G (93.5%). For FZD, combined C357T and G353A mutations were predominant, with a frequency of 100%.^[19]^ Variation in resistance mutation sites across regions provides new insights for clinical research.

VacA and CagA are 2 major virulence factors of *H pylori*. The VacA toxin can induce vacuolar degeneration in cells, leading to cellular damage and death.^[20]^ CagA promotes gastrin expression, thereby elevating the risk of peptic ulcers and gastric cancer.^[21,22]^ In this study, over 88.00% of the patients harbored both virulence factors, which may promote gastric inflammation and carcinogenesis. This further suggests that the high detection rate of these virulence factors may be associated with the high incidence of gastric cancer in the Qinghai region.

Resistance of *H pylori* to antibiotics is associated with various influencing factors. Patients over 60 years of age exhibited a significantly increased resistance rate to LVFX, which might be associated with the long-term use of LVFX for treating underlying diseases.^[23]^ Moreover, the hypoxic environment in Qinghai may affect drug metabolism in elderly patients, potentially leading to drug accumulation in the body and consequently exacerbating antibiotic resistance.^[14]^ A higher resistance rate to CLA was observed among female patients, which may be attributable to a greater propensity for healthcare-seeking behavior and higher susceptibility in women, or alternatively, to differences in hormonal profiles and gene expression.^[22]^ Meanwhile, smokers exhibited a higher resistance rate to AMX, which may be associated with nicotine-induced reduction in gastric pH or impairment of the gastric mucosal barrier during smoking, thereby decreasing drug absorption and gastric mucosal blood flow.^[24]^ This reduced drug exposure under sustained antibacterial selection pressure may predispose *H pylori* to the development of resistance. Therefore, smoking cessation is recommended prior to treatment, particularly in female smokers. Patients who frequently dine out exhibited higher TC resistance. TC is commonly used as a feed additive for animal husbandry. When humans consume animal-derived products, resistant strains may enter the human body through the food chain.^[25,26]^ Furthermore, factors such as contaminated tableware and improper food handling may increase the risk of transmission, potentially explaining the higher likelihood of resistance development among frequent diners. In contrast, none of the influencing factors had a significant impact on resistance to FZD.

Analysis of the impact of initial eradication failure on *H pylori* resistance rates, virulence, resistance genes, and resistance patterns revealed statistically significant differences in CLA and AMX resistance between the 2 groups. This indicates that antibiotic resistance may be a risk factor for treatment failure and warrants further attention. In contrast, resistance to LVFX, TC, and FZD was not significantly associated with treatment failure, although the small sample size may have influenced the statistical results. In Group B, the antibiotic resistance rates (except for FZD) and the detection rates of virulence factors were higher than those in Group A. This suggests that after initial eradication failure, increased resistance and virulence of *H pylori*, potentially due to bacterial adaptation or mutations in virulence factors, may be the core reason for treatment failure. Therefore, it is crucial to improve the 1st-line eradication success rate in clinical practice to prevent a further increase in resistance. A detailed analysis of the individual CLA and AMX resistance mutation sites revealed no statistically significant differences. Furthermore, the number of resistance gene mutation sites and resistance patterns were lower in group B than in group A. This reduction could be because antibiotic therapy preferentially clears less adaptive or more susceptible strains, whereas certain specific strains (such as those co-harboring dual virulence factors) survive and become the dominant population.

## 5. Conclusion

The persistently high rates of *H pylori* infection and virulence factor detection in Qinghai may be linked to the region’s high incidence of gastric cancer. Moreover, the overall antibiotic resistance rate exceeded the national average, with resistance to CLA and LVFX being particularly severe. Furthermore, dual and multidrug resistance frequently involves these 2 antibiotics. Therefore, the empirical use of CLA and LVFX in *H pylori* eradication therapy should be avoided in this region, and factors such as advanced age, female sex, smoking, and frequent dining out should be considered. Furthermore, in the Qinghai region, the A926G mutation was the predominant site for TC resistance, whereas synergistic combined mutations were primary for FZD resistance, providing new clues for clinical research. In recent years, precise individualized eradication of *H pylori* has garnered significant attention. Therefore, reducing antibiotic resistance rates and improving the 1st-line eradication success rate are urgent priorities. Implementing *H pylori* antimicrobial susceptibility testing using fecal samples represents an optimal solution. This study has several limitations, including its single-center design and small sample size. Moreover, the above findings are exploratory in nature and have not been adjusted for multiple comparisons; thus, the results may be subject to confounding bias and should be interpreted with caution. Further validation of these findings will require large-sample, multicenter studies and additional endoscopic biopsy confirmation.

## Author contributions

**Data curation:** Shenghui Wei, Ling Xu.

**Formal analysis:** Shenghui Wei, Xuehong Wang.

**Funding acquisition:** Xuehong Wang.

**Investigation:** Shenghui Wei, Ling Xu.

**Methodology:** Shenghui Wei, Xuehong Wang.

**Project administration:** Xuehong Wang.

**Resources:** Xuehong Wang.

**Software:** Shenghui Wei.

**Supervision:** Xuehong Wang.

**Validation:** Shenghui Wei, Xuehong Wang.

**Writing – original draft:** Shenghui Wei.

**Writing – review & editing:** Shenghui Wei, Xuehong Wang.
